# A Case of Supposed Poisoning from Daily Contact with the Gases Generated by the Combustion of Gunpowder

**Published:** 1871-05

**Authors:** E. D. Withers

**Affiliations:** Atlanta, Ga.


### Medical and Surgical Journal.

NEW SERIES.

VOL. IXB | MAY, 1871. | No. 2.

### *A Case of Supposed Poisoning, from Daily Contact with the Gases Generated by the Combustion of Gunpowder.* By E. D. WITHERS, M. D., Atlanta, Ga.

J. McCarty called to see me March 6th, complaining of being quite unwell. I learned that he was about forty years of age, by occupation a well-digger; that, until the last three months, he had led an intemperate life, but had enjoyed general good health until within a few days.

The symptoms now presenting, are oppressed respiration, tongue dry, and covered with a brown coating, slight increased temperature of the skin, left cheek, nose and ear somewhat swollen and reddened, but no marked developments.

I prescribed, Calomel, grs. vj, Dover's Powder, grs. x, a hot pediluvia at bed-time; next morning to take a dose of oil—however, he not feeling so well next morning, did not take the oil, but sent for me to see him.

I found him setting up, unable to lie down, owing to great oppression and difficulty of respiration, suffering from considerable pain, extending from the nipples to the lower border of the ribs—more intense on the right side—respiration oppressed, circulation increased, but not in proportion to the derangement of the lungs, the vesicular murmur in lower third of both lungs undistinguishable, no crepitation, the bronchial sounds louder than natural, and clearly marked. The swelling and redness of the face was now so prominent there was no mistaking the disease of the parts—erysipeletous inflammation.

The tongue being still very dry, and the skin more parched, with scanty secretions of the kidneys, I gave a powder of, Calomel, grs. ij, Ipecac, grs. j, Nitrate of Potassa, grs. x, every three hours, until three powers should be taken. Thinking it would act of itself, did not order it to be followed by a cathartic. Ordered a blister 6x6 inches to be applied over the seat of pain.

8th.—Pain in the chest abated, breathing much improved, the vesicular respiration re-established, bronchial respiration still intensified, with slight cough developed, the face very much swollen, hyperæmia excessive, skin tense and shining, pulse and tongue about the same as of yesterday. He expresses himself as being much more comfortable than on yesterday, yet he is evidently more feeble; has had no action from the bowels.

Prescribed a full dose of Sulphate of Magnesia and applied Corbolic Acid, one drachm, Glycerine, one ounce, freely and frequently, over the affected parts.

9th.—Bowels have been thoroughly evacuated; erysipeletous symptoms aggravated, with tumefaction and redness of the other eyelid, the eye being almost closed, with here and there a few tumefied, red blotches on the cheek and forehead. Several vesicles have made their appearance, with their characteristic contents.

Recognizing no good result from the carbolic acid application, and not wishing to continue it longer, I ordered the affected surface to be thoroughly painted with the Tincture of Iodine. As there had been restlessness and but little sleep, prescribed Sulph. of Morphæ in one-half grain doses, to be given at night, and repeated until the desired effect should be obtained.

10th.—No improvement in general symptoms, pulse more feeble, indisposition to take nourishment, tumefaction of the face not abated, and of a dark purple hue. The first application of Iodine being inefficient, applied it now myself, and prescribed Muriatic Tinct. of Iron, ½ ounce, Sulph. of Quinine, 3 scruples—10 drops to be taken every four hours; ordered beef soup, milk and eggs; has slight cough, with a scant, glairy, bronchial expectoration.

11th and 12th.—Condition about the same; very slight cough, and expectoration scant; same treatment continued.

13th.—Circulation feeble, the tongue and mouth extremely dry, with sordes on the teeth and lips, difficulty in protruding the tongue, owing to its dryness, and the tumefaction of the upper lip ; cough more frequent; moist bronchial rales in upper portion of lungs, more audible in the left; expectoration altered, from a thin and glairy, to a thick yellow, muco-purelent discharge.

Both eyes now closed, hyperæmia not so great, some points œdemetous, others firm, tense and unyielding.

Continued the application, and other treatment the same, with the addition of whisky every four hours.

14th.—Upon entering the room, there was every appearance of his being in "articulo mortis." Respiration about five per minute, and very labored, but not sturterous, muscular system relaxed, amd some stupor. Upon examination, I found the circulation better than the day previous, pulse fuller, and not so frequent, intellect, after being aroused, somewhat confused.

I learn from his attendants that he has been in this condition since 12 o'clock of the previous night.

The bronchial tubes and parenchyma of the lungs, very much engorged with mucous. I ascribed this unusual condition to the fact, that there had been no expectoration since the time stated above. Prescribed, in addition to previous treatment, Muriate of Ammonia and Seneka.

15th.—Condition this morning, much more comfortable. The expectorant produced the desired effect, and he has been discharging from the lungs quite freely. The erysipelas subsiding, one eye now open, and for the first time since his illness, some moisture of the tongue and mouth, with a desire to take food.

16th.—Despnœa still existing. In place of the moist rales, there now exists tubal and scibilant respiration, with crepitation at one or two points in anterior portion of both lungs. Posterially sounds less distinct, little or no disposition to cough, expectoration very scant, circulation a little more hurried, tongue more moist and cleaning off, face much improved. Treatment the same, except the continuance of the Iodine.

17th.—Condition about the same, breathing labored, and of an asthmatic character, can not lie down without much aggravation of the symptoms, circulation better, erysipeletous symptoms still improving.

18th.—No improvement ; dispnœa more distressing, moist rales principally in the upper portion of both lungs, tracheal rales very audible, and can be heard some distance; there is not the slightest disposition or effort to expel the obstructing mucous, although arrest of respiration seems imminent. On requesting him to cough, he readily throws off the mucous, which was not so tenacious or as deep a color as heretofore; his intellect is not affected, and strength remarkable for the time he has been sick.

I increased the quantity of Ammonia contained in the expectorant; no other change of treatment.

I left him about sundown, and he died about 12 o'clock, that night.

I present this case to the Academy, because the complications which existed almost from its commencement, were not only unusual, that of erysipeletous inflammation, in connection with almost complete paralysis of the breathing apparatus, early resulting in pluro-brocho-pneumonia, the former, with either of the latter, being not unusual; yet, its development with paralysis of the lungs, early resulting in such formidable complications. If such a case has ever been reported, I have no knowledge of it.

In the symptoms or resolution of the erysipelas, nothing uncommon was noticeable, it readily yielding to treatment; whilst the symptoms of the complications were novel, perplexing, and exceedingly interesting.

1. The arrest of the vesicular respiration and its sudden return, there being within twelve hours an entire exclusion of the air from the lower half of the lungs, and a complete restoration to a normal condition, when there was no evidence of any accumulation at the time of mucous, in the bronchial tubes or parenchyma of the lungs. No general symptoms of inflammation had existed sufficiently long to warrant an excess of mucous secreted. Neither was there any cough, or desire to throw it off, as is naturally the case where there is undue accumulation.

I can, therefore, only ascribe the loss of power, or action of the organs, to temporary paralysis.

On the eight day, we find this other marked and peculiar physiological condition:

Lying in a semi-comitose condition, with no decided marked debility, the respiration abnormally slow and labored, with much engorgement of the lungs, and no disposition to cough, or any manifestation of nature to relieve herself from the cause, interfering with the natural physical action of the organs.

The peculiarities existing in this case, I account for in the following manner: For several days previous to his illness, he had been daily exposed to the inhalation of carbonic acid gas, existing in the well from the powder used in blasting. After each blast, he would descend into it, and the system necessarily absorbed through the lungs and pores of the skin, much of this noxious element. We often see in some typhoid conditions, and from norcotic poisoning, the respiration abnormally slow, owing to the torpid condition of the nervous centres ; we also know that bronchitis, and other lung complications, are symptoms in typhoid fever, measles and small-pox—due to a morbid state of the blood, a poisonous material with which we are unacquainted. So, likewise, do we see violent bronchial catarrh, and exanthema of the skin, from the continued and excessive use of iodide of potassium. In this case, we have the same physiological conditions. Is it unreasonable to suppose that they were produced by the poisonous gas?

Another evidence of the blood poisoning, we see in this. There existed a very slight desire to cough, when the physical condition seemed most to demand it. Coughing is usually the effect of irritation of the air passages, an impression produced on the afferent fibres of the par vagum, which being conveyed to the medulla ablongata, gives rise to the transmission of motor impulses, to the muscles exercised in coughing. In this case, the irritation existed to a remarkable degree, but the afferent fibres failed to perform their duty.

It may be argued, that there was blood poisoning, but it does not follow that the poisoning was from the pre-supposed cause. True, an isolated case is not sufficient to establish positively this fact ; however, if this man had been exposed to some of the known causes producing those symptoms, we would unhesitatingly have attributed them to such.

Is it not possible that this agent might have produced this condition?

We are well acquainted with the deleterious properties of this gas, in its concentrated state, when inhaled; its toxical effects not alone dependent on the exclusion of oxygen, but also by some physiological property. The investigation of cause and effect, is a study of great interest and importance to every practitioner of medicine. Primary causes in many affections are so obscure that no scientific ray of light has, as yet, presented and unfolded to us their hidden mysteries. With such, we are satisfied with a course of treatment, expectant, "so-called," and are content to believe they will exist for a specified time.

Could this primary cause be known, we could then, without doubt, find remedial agents to arrest them in their incipiency.

While contemplating this case, another question has been suggested to my mind. That is, whether or no, (after a succession of battles) that depressed condition so difficult to overcome in subjects with minor injuries, which, in ordinary practice, would require scarcely any treatment, may not be, to a certain extent, caused by this agent.

I respectfully submit this case, and my conclusions, to the Academy.

